# Toward Rational Design of PFAS-Extracting Deep Eutectic Solvents: Bifunctional Architectures, Leaching Constraints, and Scalability Targets

**DOI:** 10.3390/molecules31122019

**Published:** 2026-06-09

**Authors:** Santiago Aparicio

**Affiliations:** Department of Chemistry, University of Burgos, 09001 Burgos, Spain; sapar@ubu.es

**Keywords:** PFAS, hydrophobic deep eutectic solvents, natural deep eutectic solvents, liquid–liquid extraction, solvent migration, COSMO-RS, molecular dynamics, techno-economics, life-cycle assessment, Safe-and-Sustainable-by-Design

## Abstract

Per- and polyfluoroalkyl substances (PFASs) constitute a chemically diverse family of persistent contaminants, the regulation of which is tightening rapidly in Europe and the United States. Granular activated carbon, selective ion exchange, and pressure-driven membranes remove many long-chain PFASs, but their performance is less robust for short-chain and ultrashort species, and all generate concentrated secondary waste streams. Hydrophobic deep eutectic solvents (DESs), including natural deep eutectic solvents (NADESs), have emerged as tunable liquid extractants able to concentrate PFASs into small solvent volumes that can be regenerated or coupled to destruction. This perspective differs from existing DES-PFAS reviews by converting qualitative solvent-selection arguments into a decision framework with explicit acceptance gates: broad PFAS affinity, a component-resolved non-migration specification for treated water, viscosity and mass-transfer limits, regenerability targets, and techno-economic/life-cycle benchmarking against incumbent processes. We refine the bifunctional DES design hypothesis by separating validated regimes from unresolved cases, identifying the reliability limits of COSMO-RS, molecular dynamics, and machine-learning screening, and defining tiered reporting requirements for early-stage studies. The central message is that PFAS-extracting DES should no longer be evaluated only by single-compound removal percentages; they must be judged as integrated, closed-loop treatment materials with solvent losses, regeneration stability, destruction compatibility, cost, and environmental impacts that are quantified from the outset.

## 1. Introduction and Regulatory Context

PFAS contamination has evolved from an emerging environmental concern into a binding regulatory and societal constraint with far-reaching implications for water treatment infrastructure worldwide. The chemical family encompasses over 14,000 substances [1,2] sharing the hallmark of exceptionally strong carbon–fluorine bonds (bond dissociation energy ≈ 536 kJ/mol [3]) that confer remarkable thermal, chemical, and biological persistence—properties that, once valued for industrial applications ranging from non-stick coatings to aqueous film-forming foams, now constitute the defining challenge for environmental remediation.

**Regulatory drivers.** In the European Union, the recast Drinking Water Directive (EU) 2020/2184 [4,5] introduced two PFAS-specific parameters that entered into force in January 2026: ‘PFAS Total’ (≤0.50 μg/L, encompassing the sum of all measurable PFAS) and ‘Sum of PFAS’ (≤0.10 μg/L, defined as the sum of 20 specified compounds spanning C4–C13 perfluoroalkyl carboxylates and sulfonates). In the United States, EPA’s April 2024 National Primary Drinking Water Regulation [6] established individual MCLs of 4 ng/L each for PFOA and PFOS, 10 ng/L each for PFHxS, PFNA, and HFPO-DA (GenX chemicals), and a hazard-index mixture approach for the combined occurrence of four regulated PFAS. These convergent regulatory instruments, coupled with mounting evidence of widespread trifluoroacetic acid (TFA) contamination at μg/L levels in European surface waters [7,8] and the European Chemicals Agency’s proposed broad PFAS restriction covering approximately 10,000 substances [9], are compelling utilities to urgently evaluate advanced treatment and source-control strategies that extend well beyond the capabilities of conventional water treatment plants.

**Incumbent technologies: capabilities and gaps.** The three technologies designated as Best Available Technologies by the US EPA for PFAS removal [6,10] are GAC adsorption, PFAS-selective anion exchange (IX), and high-pressure membranes (RO/NF). GAC is the most widely deployed, operating at empty bed contact times of 10–20 min [10,11], but its efficacy declines sharply for short-chain perfluorocarboxylic acids (C4–C6), and competitive adsorption by natural organic matter (NOM) can reduce effective bed life by 30–70% [11,12]. Selective IX resins achieve substantially longer operational capacities (100,000–400,000 bed volumes versus 10,000–50,000 for GAC [10,13]) with correspondingly smaller system footprints, but at an approximately three-fold higher unit media cost, and their regeneration remains technically challenging. RO/NF achieves near-complete PFAS rejection across all chain lengths, but at substantial energy cost (0.5–1.5 kWh/m^3^ [10]), produces a PFAS-laden concentrate requiring further treatment, and is impractical for many small- and medium-sized utilities. Critically, none of these technologies destroys PFASs; they merely sequester or concentrate these persistent contaminants, thereby deferring the ultimate disposal challenge to downstream processes, typically incineration under conditions that may not achieve complete defluorination [14,15].

**Deep eutectic solvents as an alternative paradigm.** Deep eutectic solvents are structured liquids formed by complexation of a hydrogen bond acceptor (HBA) with one or more hydrogen bond donors (HBDs), yielding pronounced melting-point depressions and liquids with tuneable polarity, viscosity, and interfacial properties. First introduced using choline chloride–urea mixtures by Abbott et al. in 2003 [16], the DES concept was extended to hydrophobic, water-immiscible formulations in 2015 by van Osch et al. [17]. Only recently has this conceptual framework been explicitly translated to PFAS removal. The trajectory of progress has been rapid: proof-of-concept work using a menthol:acetic acid NADES achieved extraction of perfluoroheptanoic acid (PFHpA) [18] but simultaneously revealed a critical limitation—partial leaching of the acidic component into the treated water; COSMO-RS-guided multi-criteria screening subsequently identified natural hydrophobic DES candidates for PFOA and PFOS [19,20] with improved phase stability; integrated experimental–computational work then demonstrated near-quantitative PFOA removal with excellent reusability for a TOPO:lauric acid DES [21]; and most recently, an energy-based screening strategy achieved [22] >99% removal efficiency across multiple PFAS classes—including perfluorocarboxylic acids (PFCAs), perfluorosulfonic acids (PFSAs), and perfluoroalkyl amides—in real water matrices [22,23]. In an important conceptual advance, hydrophobic DES platforms have now been demonstrated to be capable of integrated PFAS capture and in situ mild-condition mineralization [24], representing a paradigm shift from separation-only to separation-plus-destruction architectures.

**Scope of this perspective.** This perspective is organized around four non-negotiable design targets: (1) high extraction affinity across both long-chain and short-chain PFAS (including the full EU PFAS-20 list and emerging ultrashort species such as TFA); (2) quantitative non-migration specifications enforced at environmentally relevant concentrations (ng/L–μg/L); (3) exclusive reliance on natural, benign, biodegradable, and low-cost components compatible with large-scale industrial supply chains; and (4) demonstrated process scalability at total treatment costs competitive with or below those of incumbent technologies (<$0.50/m^3^ for community-scale systems). We deliberately prioritize molecular design principles, quantitative decision criteria, and techno-economic benchmarks over exhaustive cataloguing of DES candidates—an approach we believe offers greater translational value at this stage of the field’s development.

**Figure 1:**
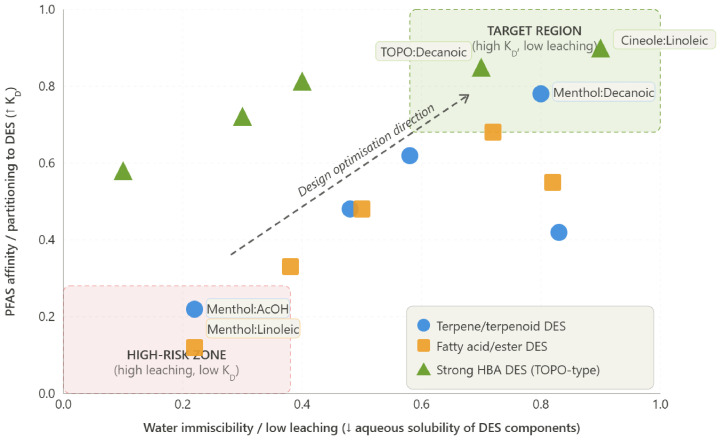
Conceptual design map for PFAS-extracting DES. The vertical axis represents increasing PFAS affinity, ideally expressed experimentally as log KD for a defined PFAS mixture, while the horizontal axis represents decreasing water miscibility and decreasing component-resolved leaching risk. The shaded target region is therefore not proof that any specific family is universally superior; it identifies the simultaneous performance space that must be experimentally demonstrated. Candidate locations should be interpreted as hypothesis-driven positions based on available solubility, polarity, and PFAS-partitioning trends, not as a high-throughput experimental ranking.

**Positioning of this article.** This manuscript is a critical perspective rather than an exhaustive review or a primary experimental report. Its purpose is therefore not to rank all published DES formulations, but to define a falsifiable design-and-validation roadmap for PFAS-extracting DES, Figure 1. We now distinguish explicitly between mechanisms that are experimentally supported, targets that are engineering specifications, and hypotheses that require further validation. This revision also clarifies the operational meaning of the screening gates so that readers can decide when a candidate should advance, be reformulated, or be rejected.

## 2. Molecular Design Rules and Validation Strategy

### 2.1. Molecular-Level Requirements for PFAS Extraction

**Figure 2:**
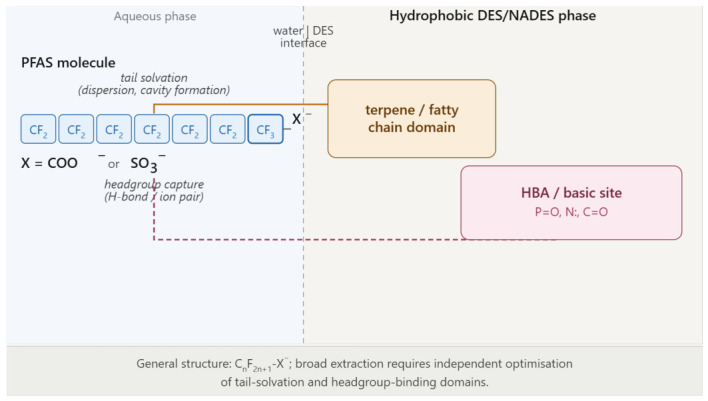
Bifunctional DES extraction concept and general molecular interaction motif. The DES phase provides two complementary domains: a hydrophobic terpene/fatty-acid-like domain that solvates the perfluoroalkyl tail through dispersion interactions and cavity formation, and a headgroup-binding domain containing hydrogen-bond-accepting or basic motifs (e.g., P=O, C=O, or amine/amide sites) that stabilize carboxylate, sulfonate, amide, or precursor headgroups through hydrogen bonding, ion pairing, or electrostatic interactions. The new panel emphasizes that broad-spectrum extraction requires simultaneous optimization of tail solvation, headgroup capture, and water—DES interfacial transfer.

PFASs are surface-active amphiphiles comprising a highly lipophobic and oleophobic perfluoroalkyl tail (C^n^F^2n+1^–) coupled to a polar headgroup that may be anionic (carboxylate and sulfonate), zwitterionic, or neutral (amides, fluorotelomer alcohols, and precursors). This fundamental dual characteristic imposes simultaneous and conflicting requirements on any liquid extractant: (i) a strong thermodynamic driving force for transferring the fluorinated tail out of the aqueous phase, quantifiable through the excess chemical potential contribution Δμ^ex^ for the tail fragment, and (ii) specific non-covalent interactions with the polar headgroup that persist in a low-dielectric, structured liquid environment where water molecules are largely absent. DESs offer two independently tunable molecular levers with which to satisfy both requirements: the bulk polarity and nanostructure of the HBA–HBD hydrogen-bond network, and the interfacial activity and self-assembly behaviour of the individual components at the water–DES boundary [25,26], Figure 2.

### 2.2. Bifunctional DES Design Hypothesis

We propose that broad-spectrum PFAS extraction is most robustly achieved when the DES provides two complementary and independently optimizable interaction motifs—a principle we term the ‘bifunctional DES hypothesis’:

**Hydrophobic domain (tail solvation).** A bio-derived hydrophobe—such as a monoterpene (menthol, thymol, 1,8-cineole), a terpenoid ester, or a medium-chain fatty acid (C8–C14)—generates a low-polarity microenvironment thermodynamically favourable for the perfluoroalkyl chain. The key molecular descriptor governing efficacy is the infinite-dilution activity coefficient of the perfluoroalkyl fragment in the DES phase (γ^∞^tail, DES), which must be significantly lower than the corresponding value in water. COSMO-RS calculations consistently demonstrate that terpene-rich DES achieve [19,20,27] γ^∞^ values 2–4 orders of magnitude lower than water for C6–C12 perfluoroalkyl chains, providing the requisite thermodynamic driving force for extraction.

**Headgroup-binding domain (polar capture).** A strong hydrogen-bond acceptor that stabilizes the protonated acid form of PFAS (for operation below the pK_a_, typically 0–2 for PFCAs) or a basic/ionizable site forms tight ion pairs with PFAS anions (for operation at environmental pH, 6–8). In practice, this requires incorporating a moiety with high Abraham hydrogen-bond basicity (β > 0.8), such as tertiary amine oxides, long-chain amides, or phosphine oxides. The demonstration of PFAS extraction by TOPO-based DES elegantly illustrates the potency of strong HBAs [21]; however, TOPO is not a ‘natural’ component under current Safe-and-Sustainable-by-Design frameworks, motivating the search for bio-based functional analogues, including matrine (a tetracyclic quinolizidine alkaloid from *Sophora flavescens* [28]), phospholipid-derived phosphine oxides, or sterol-derived amine oxides.

The bifunctional concept is strongly supported by recent ab initio molecular dynamics (AIMD) simulations [23], which demonstrate that DES components self-organize to form a complementary, flexible non-covalent interaction network around diverse PFAS targets, with spatially distinct solvation shells for the fluorinated tail and the polar headgroup operating simultaneously within the nanostructured liquid.

**Experimental anchoring of the design principle.** A fully matched pair comparing a purely hydrophobic DES and an otherwise identical bifunctional DES for a complete PFAS mixture has not yet been reported; this is now stated explicitly as an important data gap. Nevertheless, the existing literature provides directional support: simple menthol/short-acid NADES demonstrated the feasibility of hydrophobic extraction but also revealed leaching limitations [18], whereas TOPO:lauric acid and ionizable DES platforms showed stronger PFOA or multi-PFAS uptake when a high-basicity headgroup-binding motif was present [21,22,23]. The revised text, therefore, presents the bifunctional concept as a testable design hypothesis supported by convergent evidence, rather than a universally proven mechanism.

**Validated and unresolved regimes.** The bifunctional hypothesis is best supported for long-chain and mid-chain PFCAs/PFSAs (approximately C6–C12), for which hydrophobic tail transfer provides a large thermodynamic driving force and strong HBA/basic motifs can stabilize the polar headgroup. It is partially supported for C4–C6 short-chain acids, neutral precursors, and amide-like PFAS, where headgroup-specific interactions become more decisive, and the role of ionic strength increases. It remains a working hypothesis, not an established mechanism, for ultrashort PFAS such as TFA and PFPrA, the hydration free energies, small hydrophobic volume, and high mobility of which make extraction much less likely to be governed by the same tail-solvation term. These domains are now treated separately throughout the screening workflow.

### 2.3. Non-Migration as a First-Class Design Objective

Solvent loss to the aqueous phase is the central technical barrier for translating DES extraction into potable-water or discharge-quality treatment. From a regulatory and risk perspective, it is wholly insufficient to demonstrate ‘phase separation’ macroscopically or to report a low percent mass loss: the operationally relevant targets are component-resolved concentrations at ng/L–µg/L levels in the treated water, assessed using analytical methods with appropriate limits of quantification. We therefore advocate for a formal ‘non-migration specification’ analogous to the specific migration limits established for food-contact polymers under EU Regulation (EU) No 10/2011 [29], encompassing three quantitative criteria: (a) each individual DES component must exhibit an aqueous solubility below a defined threshold (e.g., <10 mg/L at 20 °C; operational target <1 µg/L per component in the effluent); (b) no formation of water-soluble complexes between DES components and PFAS, NOM, or inorganic matrix constituents; and (c) no pH drift exceeding ±0.3 units in the treated water after extraction. The <1 µg/L per component value is not presented as a current legal limit for DES constituents; it is a conservative engineering target proposed by analogy with migration-control frameworks and the analytical expectations of trace-contaminant drinking-water treatment.

**Mechanistic drivers of migration risk.** Leaching risk increases substantially when: (i) one component is a small, water-miscible carboxylic acid (as in the prototypical menthol:acetic acid DES, where the aqueous solubility of acetic acid is effectively infinite [18]); (ii) the DES contains hydrotropic or surfactant-like molecular fragments that promote emulsion formation at the phase boundary; or (iii) stable microemulsions form in the presence of NOM, co-extracted surfactants, or divalent cations. To suppress migration, the component library must be deliberately biased toward larger, hydrophobic, bio-based molecules—C8–C14 fatty acids or their esters, bicyclic terpenes and terpenoids, long-chain amides—and decisively biased away from short-chain acids, alcohols, and phenols with appreciable water solubility. When ionic or ionizable characteristics are required for PFAS anion capture, the ionizable motif should be ‘masked’ within an overall hydrophobic scaffold (e.g., bulky tertiary amines with calculated log*p* > 3) rather than introduced as a water-soluble salt or small polar molecule.

### 2.4. Quantitative Design Criteria

Table 1 consolidates the minimum quantitative criteria that a PFAS-extracting DES must satisfy, organized according to the four design targets of this perspective, as well as illustrative examples in Table 2. The values should be read as transparent go/no-go gates, not as claims that have already been achieved simultaneously. The table therefore distinguishes three evidence classes: criteria demonstrated in at least one literature system, criteria partially demonstrated for restricted PFAS classes or cycle numbers, and prospective engineering targets derived from incumbent-technology benchmarking, migration-control logic, or TEA/LCA constraints.

### 2.5. Predictive Modelling: Three Sequential Computational Gates

**Gate 1—DES formation and phase behaviour.** Gate 1 predicts the solid–liquid equilibrium to identify compositions that yield a stable liquid phase at the target operating temperature (T < 25 °C preferred for ambient water treatment). COSMO-RS provides rapid estimation of the excess Gibbs energy of mixing [30,31] (ΔG^E^*mix*) and component activity coefficients for candidate HBA–HBD pairs, enabling screening of thousands of combinations within hours. A recognized limitation is the treatment of solid-state contributions to the melting-point depression; we recommend calibrating predictions against a minimum of five experimentally characterized DES per structural family.

**Gate 2—Water immiscibility and component aqueous solubility.** Gate 2 predicts the mutual solubility of each DES component in water at operational pH and ionic strength. COSMO-RS is well-suited for this prediction, although calculated aqueous solubilities for terpenes and fatty acids may deviate by up to one order of magnitude from experimental values. Molecular dynamics simulations with explicit hydration shells provide complementary and often more accurate validation for the most promising lead candidates.

**Gate 3—PFAS partitioning and selectivity.** Gate 3 predicts distribution coefficients (K_D_) for the complete target PFAS set. COSMO-RS enables rapid screening of thousands of DES–PFAS combinations and has already been applied successfully for multi-criteria selection of hydrophobic DES for PFOA and PFOS [19,20]. However, COSMO-RS may systematically underestimate specific ion-pairing contributions and interfacial accumulation effects that are particularly important for short-chain sulfonates. Targeted classical and ab initio molecular dynamics simulations validate binding motifs, quantify hydration-shell disruption energetics, and probe sensitivity to counter-ion identity and salinity. We strongly recommend that competitive uptake of NOM surrogates (e.g., Suwannee River humic and fulvic acid molecular fragments) be explicitly included in the modelling protocol, as NOM frequently dominates capacity losses and selectivity degradation in real water matrices [11,12].

**Machine-learning opportunity and data gap.** ML can accelerate DES discovery, but the DES|PFAS domain does not yet have a large, standardized database comparable to those available for conventional solvent properties. Existing ML studies have demonstrated useful descriptors for DES formation and contaminant removal [32,33], yet PFAS-specific models should be trained through active learning with experimentally measured KD, viscosity, water uptake, component leaching, toxicity flags, and cost. Until such FAIR datasets are available, ML outputs should be used to prioritize experiments and define Pareto fronts, not to replace component-resolved validation.

**Recommended simulation scale.** The revised workflow now uses a tiered computational organization: (i) COSMO-RS or equivalent thermodynamic screening for hundreds to thousands of candidates; (ii) classical MD of 3–10 shortlisted systems using explicit biphasic water|DES slabs, realistic salinity, and at least one NOM surrogate over approximately 50–200 ns; and (iii) AIMD only for the most uncertain binding motifs, typically 100–300 atoms and 10–30 ps trajectories. This scale is sufficient to test whether predicted headgroup binding and tail solvation are chemically plausible, while avoiding the unrealistic expectation that AIMD can sample complete extraction equilibria.

**Practical reliability domain.** COSMO-RS should be treated as a high-throughput triage tool for neutral or weakly ionized components, relative hydrophobicity, DES formation tendencies, and first-order partitioning trends within structurally similar families. Its high-uncertainty domain includes strongly ion-paired PFAS, interfacially accumulated species, short-chain sulfonates, the extraction of which is dominated by counter-ions and hydration-shell disruption, and real waters where NOM, divalent cations, or co-surfactants control phase behaviour. Classical MD is more reliable for explicit water|DES interfaces, diffusion, aggregation, and NOM competition, but depends strongly on force-field quality for fluorinated tails and ionizable DES components. AIMD is most valuable for short mechanistic validation of hydrogen bonding, proton transfer, and ion-pair motifs; it should not be used as a brute-force screening method.

### 2.6. Process Architectures for Non-Migration and Scalability

**Figure 3:**
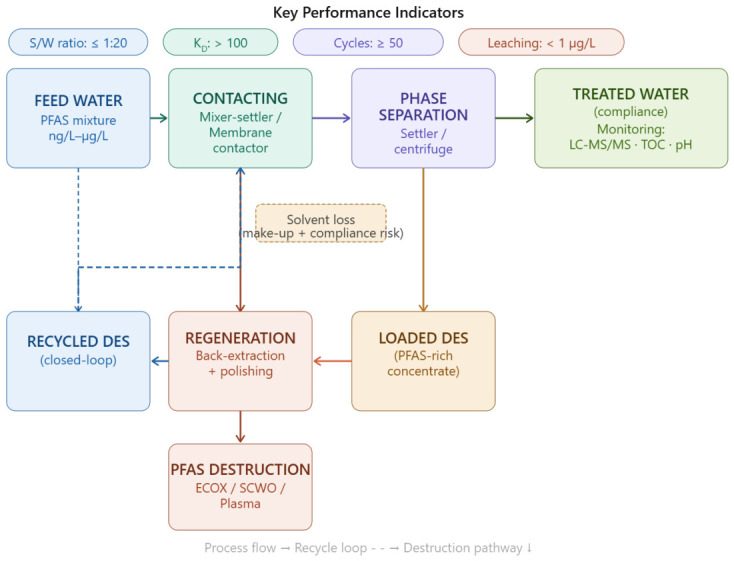
Closed-loop DES extraction process concept comprising six principal unit operations: feed water contacting (mixer–settler or membrane contactor), phase separation, treated water discharge with compliance monitoring, loaded DES handling, regeneration (back-extraction, polishing, and quality verification), and solvent recycle. Key performance indicators are annotated at the top. PFAS destruction is integrated as a dedicated module receiving the concentrated waste stream from regeneration. Monitoring points for component-resolved leaching (LC-MS/MS), TOC drift, and pH drift are indicated.

**The extraction process based on DES, Figure 3, has several relevant features, which are analyzed sequentially.**

**Classical liquid–liquid extraction.** Mixer–settlers or centrifugal contactors (e.g., Cinc units) offer rapid phase separation (<10 s residence time), high specific throughput, and compact footprint—characteristics well-established in the hydrometallurgical and petrochemical industries. However, direct phase dispersion maximizes the DES–water interfacial area, inherently increasing the risk of solvent carryover through entrainment and micro-emulsion formation, particularly in waters with elevated NOM.

**Membrane contactors.** Hollow-fibre membrane contactors decouple mass transfer from phase dispersion: the aqueous feed and DES phases flow on opposite sides of a microporous or dense polymeric membrane, with PFAS transferring diffusively across the stabilized interface. This architecture is particularly attractive for potable-water applications because it eliminates droplet entrainment and reduces solvent carryover to diffusion-limited losses through the membrane material. The principal trade-offs are susceptibility to membrane fouling by NOM or colloidal matter and the incremental capital cost of membrane modules.

**Integrated capture-and-destroy architectures.** The most forward-looking process concept integrates PFAS extraction with sequential or in situ destruction. Recent work has demonstrated that PFAS concentrated in a hydrophobic DES phase can be mineralized under remarkably mild conditions [24]—ambient pressure and temperatures below 80 °C—using electrochemical oxidation, UV/persulfate activation, or Fenton-like systems operating directly within the DES medium. This approach eliminates the back-extraction step entirely, potentially simplifying the overall process train and reducing the total number of unit operations.

**Regeneration is the decisive cost driver.** For process architectures requiring DES recycling, the simplest and most scalable regeneration route involves back-extraction of PFAS into a small volume of alkaline brine (NaOH 0.1–1 M in a methanol/water cosolvent) followed by destruction of the concentrated PFAS-rich eluate by electrochemical oxidation, non-thermal plasma, or supercritical water oxidation—operating on a waste volume 100–1000× smaller than the original feed. The stripped DES phase, after analytical verification of composition integrity by NMR, FT-IR, and viscometry, is returned to the contacting stage to begin a new extraction cycle.

**Example of a DES-compatible treatment train.** For terpene/fatty-acid or alkaloid-containing hydrophobic DES, the lowest-risk near-term option is not direct oxidation of the bulk DES but closed-loop regeneration followed by destruction of a small aqueous/alcoholic concentrate. A practical sequence is as follows: alkaline methanol/water back-extraction (0.1–1 M NaOH), recovery of methanol for reuse, electrochemical oxidation of the concentrated PFAS brine using a boron-doped-diamond or mixed-metal-oxide anode, and confirmation of mineralization by fluoride release, total organic fluorine closure, LC-HRMS screening of intermediates, and residual DES-component analysis. SCWO is a more aggressive alternative for the concentrate when complete mineralization is prioritized, but it should be applied to the stripped concentrate rather than to the full DES inventory unless solvent sacrifice is acceptable.

## 3. Techno-Economic and Life-Cycle Assessment Framework

A rigorous techno-economic analysis (TEA) of DES-based PFAS extraction requires identification and quantification of the principal cost levers that distinguish this approach from established adsorption and membrane technologies. We identify six primary cost drivers that collectively determine the economic competitiveness of DES extraction systems, and for each, we establish indicative target ranges derived from benchmarking against community-scale GAC and IX installations serving populations of 1000–100,000 (Table 3).

**Solvent acquisition and make-up cost.** The unit cost of DES components (€/kg) and the fractional solvent loss per extraction cycle (% per cycle) are tightly coupled parameters that together constitute the single most influential variable in the total OPEX equation. Bio-based terpenes (menthol and thymol) currently trade at €8–25/kg in bulk, while medium-chain fatty acids (octanoic, decanoic, and lauric acid) are available at €2–8/kg. At a solvent-to-water ratio of 1:20 (*v*/*v*) and a solvent make-up rate of 1% per cycle, the solvent replacement cost alone amounts to approximately $0.005–0.015/m^3^—an acceptably small fraction of total OPEX. However, if make-up rates escalate to 5–10% per cycle due to entrainment, emulsification, or chemical degradation, solvent cost can rapidly dominate the cost structure and render the technology uncompetitive. This underscores the critical importance of the non-migration specification (Section 2.3) as an economic requirement, not merely an environmental one.

**Contactor and phase-separation capital costs.** The choice of contacting architecture profoundly affects both CAPEX and achievable solvent losses. Conventional mixer–settlers, while proven and scalable, require a substantial footprint and may entrain DES at levels exceeding potable-water specifications. Centrifugal contactors (e.g., Cinc^®^, Rousselet Robatel) offer 10–100× volumetric intensification but at a higher unit equipment cost ($15,000–80,000 per stage). Hollow-fibre membrane contactors present the lowest entrainment risk [34] and are available as modular commercial units (Liqui-Cel^®^, 3M), with demonstrated applicability to solvent-extraction processes in the pharmaceutical and fine-chemical industries. For a 1 MLD (mega-litre per day) treatment plant, we estimate that the membrane-contactor CAPEX would add $0.03–0.08/m^3^ on an annualized basis (20-year lifetime; 5% discount rate), which is a premium offset by reduced solvent losses and simplified downstream polishing.

**Regeneration and destruction costs.** The energy and chemical inputs required for solvent regeneration and PFAS destruction represent the second-largest OPEX component. Back-extraction into alkaline methanol/water brine [21,24] (NaOH 0.1–1 M) followed by electrochemical oxidation of the concentrated eluate has been estimated at $0.02–0.08/m^3^ of original feed water, assuming a concentration factor of 200–500× between the feed and regeneration eluate. Supercritical water oxidation (SCWO) of the concentrated stream, while achieving near-complete mineralization of both PFAS and any co-extracted DES residues, carries higher energy penalties ($0.05–0.15/m^3^) but eliminates concerns about incomplete degradation by-products. Non-thermal plasma treatment of the loaded DES phase is an emerging alternative with a cost structure that remains poorly characterized; we identify this as a priority area for techno-economic data generation.

**Analytical monitoring costs.** A frequently overlooked but operationally significant cost component is the analytical monitoring programme required to verify both PFAS removal performance and DES migration compliance. Unlike GAC or IX systems, where routine monitoring focuses solely on PFAS breakthrough in the treated water, DES-based systems require parallel monitoring of DES component concentrations in the effluent, typically by LC-MS/MS with dedicated analytical methods achieving LOQ ≤ 50 ng/L per component. We estimate that this adds $0.02–0.05/m^3^ to operational costs, depending on sampling frequency and the availability of multi-analyte methods. Development of rapid, field-deployable screening methods (e.g., immunoassay-based or fluorescence-based sensors for terpene/fatty acid residues) could substantially reduce this cost burden and should be pursued as a parallel research priority.

**Benchmarking concentration regime.** The GAC and IX cost ranges used here correspond primarily to ng/L to low-µg/L drinking-water or groundwater matrices, where large treated volumes and low influent concentrations dominate economics. They should not be extrapolated directly to high-concentration industrial effluents, landfill leachates, or AFFF-impacted concentrates, for which brine management, co-contaminants, and destruction costs may be more important than polishing to drinking-water limits.

**Cost-window sensitivity.** The sub-$0.50/m^3^ target is feasible only under a narrow but transparent set of assumptions: solvent make-up below approximately 2% per cycle, regeneration/destruction below about $0.10/m^3^ of feed, and monitoring costs maintained near $0.02–0.05/m^3^ through multi-analyte methods or regionalized testing. Two parameters most readily break the cost window: solvent loss above 5% per cycle, which can make solvent replacement the dominant OPEX, and frequent bespoke LC-MS/MS monitoring above $0.10/m^3^. Conversely, industrial effluents with higher PFAS concentrations may tolerate higher unit costs if the DES process replaces off-site disposal or enables destruction of a much smaller concentrate.

Table 3 synthesizes these cost levers into a comparative framework against GAC and IX benchmarks, providing quantitative targets that DES research should aim to meet or exceed.

### Life-Cycle Assessment: Requirements, Hotspots, and Reporting Standards

Published LCAs of PFAS treatment technologies have demonstrated that environmental impacts are distributed unevenly across the process chain: energy consumption for pumping and pressure generation, manufacturing and transport of consumable media (activated carbon or ion-exchange resin), and management of spent media or concentrated waste streams typically dominate across multiple impact categories, including climate change, cumulative energy demand, and ecotoxicity. For DES-based extraction, the environmental impact profile differs structurally from adsorption-based technologies and introduces four distinct and potentially significant additional hotspots that must be quantified:

(i) **Solvent synthesis and purification.** Even when individual DES components are sourced from renewable feedstocks, their extraction, purification, and formulation into a binary or ternary eutectic mixture may carry substantial embedded energy and environmental burdens. Menthol production from *Mentha arvensis*, for example, involves agricultural land use, solvent extraction, fractional distillation, and crystallization—unit operations with a cumulative impact per kilogram that may rival or exceed that of petroleum-derived solvents. Synthetic menthol from thymol or citral, while avoiding direct agricultural land use, has its own petrochemical supply chain implications. A rigorous LCA must trace the complete cradle-to-gate pathway for each component, including agricultural inputs (fertilizers, pesticides, and water) where applicable.
(ii) **Solvent losses to the aqueous and atmospheric phases.** Aqueous losses directly affect both the environmental quality of the treated water (addressed by the non-migration specification) and the mass balance of solvent that must be replaced. Atmospheric losses are relevant for volatile DES components, particularly terpenes with vapour pressures of 0.01–0.1 kPa at ambient temperature. These emissions contribute to photochemical ozone creation potential (POCP) and may trigger air quality permitting requirements at larger scales. Enclosed contacting systems (membrane contactors and sealed centrifugal extractors) mitigate both loss pathways simultaneously and should be strongly preferred in the LCA boundary design.
(iii) **Regeneration chemicals and energy.** The alkaline brine or cosolvent used for PFAS back-extraction (NaOH, methanol) must be accounted for in the LCA inventory, as must the energy inputs for any thermal regeneration steps. Methanol recovery by distillation, if employed, adds an energy-intensive unit operation, the impact of which scales with the regeneration volume and frequency. Life-cycle impacts of the destruction step for the concentrated PFAS eluate (electrochemical oxidation, SCWO, and plasma) are highly technology-specific and remain poorly characterized in the current literature; we identify this as a critical data gap.
(iv) **Compatibility of loaded DES with destruction methods.** When PFAS is destroyed within or from the DES matrix, the organic DES components themselves may undergo partial degradation, potentially forming toxic or persistent by-products. This risk is particularly acute for high-energy destruction methods (SCWO, plasma) operating at conditions that may also mineralize or fragment the solvent. A complete LCA must therefore include emissions and by-product characterization from the destruction stage, not merely the PFAS defluorination efficiency.

We propose that all publications reporting DES-based PFAS extraction include, at minimum, three environmental impact indicators normalized per m^3^ of water treated: (1) climate change (kg CO_2_-eq/m^3^); (2) cumulative energy demand (CED, MJ/m^3^); and (3) at least one toxicity-related endpoint (freshwater ecotoxicity potential or human toxicity potential), alongside a composite ‘residual risk’ metric that captures both PFAS and solvent traces remaining in the treated water. Where full ISO 14040/44-compliant LCA [35] is not feasible at the current early research stage (TRL 2–3), researchers should at minimum compile a complete life-cycle inventory comprising all mass and energy inputs and outputs for the extraction, regeneration, and destruction stages, thereby enabling subsequent harmonization and meta-analysis as the field matures.

We emphasize that ‘natural’ is not synonymous with ‘low environmental impact’ [36,37]—a conflation that pervades the green-chemistry literature and risks undermining the credibility of DES-based approaches. Bio-based components sourced from land-intensive monoculture agriculture, requiring energy-intensive purification steps, or exhibiting poor aquatic biodegradation kinetics may well carry higher life-cycle impacts than carefully selected synthetic alternatives. Component sourcing, production efficiency, number of reuse cycles achieved in practice, and end-of-life management must all be quantified explicitly within the LCA system boundary and reported transparently.

**Impact hotspot for bio-based precursors.** For DES components sourced from crops rather than residues, the dominant impact may shift from solvent use during extraction to fertilizer production, field emissions, irrigation water demand, and biodiversity pressure. We therefore added explicit guidance that any claim of environmental superiority must report feedstock origin, allocation method, purification yield, number of reuse cycles, and degradation/end-of-life assumptions.

**Concrete supply-chain example.** Menthol illustrates why the ‘natural equals green’ assumption can fail. Menthol isolated from dedicated Mentha arvensis cultivation may carry burdens from irrigation, fertilizer and pesticide inputs, land occupation, steam distillation, and fractional purification. A chemically equivalent or functionally similar terpene component produced from waste-derived limonene/citral streams from citrus processing could have lower land-use and agricultural burdens, even if it involves additional catalytic conversion steps. The LCA conclusion can therefore invert the intuitive preference for the directly plant-derived component once allocation, purification yield, transport, and solvent lifetime are included.

**Figure 4:**
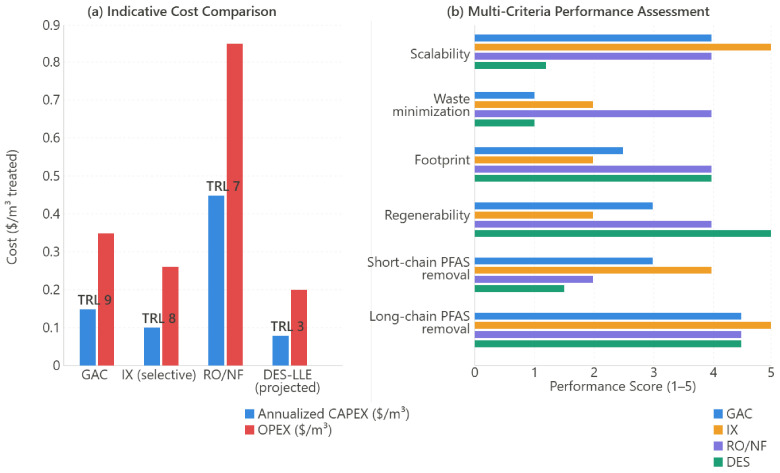
Benchmarking DES-based PFAS extraction against incumbent technologies. (**a**) Indicative cost comparison showing annualized CAPEX and OPEX per m^3^ treated for GAC, selective IX, RO/NF, and projected DES-LLE, with Technology Readiness Level (TRL) annotated for each. (**b**) Multi-criteria performance assessment across six dimensions on a 1–5 scale. DES-LLE shows competitive projected performance in PFAS removal efficiency and waste minimization but requires significant advancement in demonstrated scalability (current TRL 3).

Finally, a benchmarking of DES-based extraction technologies is reported in Figure 4, which serves as a suitable approach to analyze the realistic scenarios for the scaling-up of the considered technologies.

## 4. Strategic Assessment: SWOT and PESTLE Analysis

Translating a novel separation technology from laboratory proof-of-concept to commercial deployment requires not only demonstrated scientific merit but also a clear-eyed assessment of competitive positioning relative to incumbent technologies, regulatory compatibility within existing and anticipated frameworks, supply-chain robustness, and societal acceptance. The following SWOT (Section 4.2) and PESTLE (Section 4.1) analyses provide a structured strategic evaluation of DES-based PFAS extraction at its current stage of development (TRL 2–3), informed by the techno-economic and LCA framework developed in Section 3 and by analogy with the adoption trajectories of other novel water treatment technologies that have successfully (or unsuccessfully) traversed the path from laboratory to practice.

Application-specific interpretation. The strategic risks are not identical for potable water and industrial effluents. For potable water, the decisive issues are public acceptance, trace-level solvent migration, regulatory approval of every DES component, and reliable operation at ng/L PFAS concentrations. For industrial effluents, landfill leachates, or AFFF-impacted side-streams, the opportunity is greater because influent PFAS loads are larger and pre-concentration has immediate value, but the dominant challenges shift toward matrix robustness, co-extraction of surfactants and organics, regeneration/destruction of high-load concentrates, and worker-safety controls. The revised workflow, therefore, treats drinking-water deployment as the most stringent endpoint, while recognizing industrial side-streams as more realistic first demonstration markets.

### 4.1. PESTLE Analysis

The PESTLE framework (Political, Economic, Social, Technological, Legal, Environmental) systematically examines the external macro-environmental factors that will shape the adoption trajectory of DES-based PFAS extraction over the next 5–10 years. Table 4 presents a detailed assessment of each factor alongside recommended strategic responses.

**Political factors.** The current political landscape is overwhelmingly favourable for PFAS treatment innovation. The EU Drinking Water Directive (2020/2184) entered full force in January 2026 with legally binding PFAS limits, creating immediate compliance demand across approximately 100,000 water supply zones in Europe [4,5]. In the United States, the April 2024 NPDWR established enforceable PFAS standards for the first time [6], with public water systems required to complete initial monitoring within three years. Concurrently, the EU’s proposed universal PFAS restriction—potentially the most extensive chemical regulation in history—signals a long-term policy trajectory toward comprehensive PFAS phase-out that will sustain and expand treatment demand for decades. Critically, political funding programmes (Horizon Europe, LIFE, US EPA Water Innovation grants) increasingly prioritize technologies that are both deployable and demonstrably lower risk than incumbents, creating both opportunity and constraint for novel approaches like DES extraction.

**Economic factors.** The economic landscape presents both compelling opportunities and significant challenges. Small and medium-sized water utilities (serving populations <10,000) face disproportionately high per capita compliance costs because incumbent technologies exhibit strong economies of scale that disadvantage smaller systems. DES-based extraction, if implemented as modular containerized units requiring minimal site infrastructure, could directly address this market segment. However, the economic case is sensitive to two parameters that remain poorly quantified: (i) solvent make-up cost over extended operational periods (months to years), and (ii) the cost of the dedicated analytical monitoring programme required to verify both PFAS removal and DES migration compliance. For DES extraction to achieve economic competitiveness, the total treatment cost (CAPEX + OPEX including monitoring) must fall below $0.50/m^3^ for systems serving >3000 population equivalents—a target that appears achievable on paper but remains to be demonstrated at pilot scale.

**Social factors.** Public perception of water treatment additives presents a distinctive challenge for DES-based extraction that does not affect adsorption-based technologies. Whereas GAC and IX are perceived as ‘filtering’ technologies that remove contaminants, liquid–liquid extraction introduces a chemical agent into intimate contact with drinking water—a distinction that may trigger public concern regardless of the actual risk profile. The use of food-grade, GRAS-listed (Generally Recognized as Safe) components—many of which are already present in consumer food products as flavourings or preservatives—provides the strongest available foundation for social acceptance. Transparent publication of third-party toxicological data, aquatic ecotoxicity assessments, and biodegradability results in open-access venues is essential for building and maintaining social licence.

**Technological factors.** Several unresolved technological challenges define the current development bottleneck. Mass-transfer limitations imposed by the characteristically high viscosities of many DES formulations (50–500 mPa·s at 25 °C, compared to 0.5–2 mPa·s for conventional organic solvents) may require elevated operating temperatures (40–60 °C), prolonged contact times, or intensive mechanical agitation—all of which add to energy costs and process complexity. Fouling of membrane contactors by NOM, colloidal matter, and biofilm formation in real water matrices remains uncharacterized for DES systems. Validated regeneration protocols demonstrating stable extraction performance over industrially relevant cycle numbers (≥50 cycles, ideally >200) are entirely lacking for PFAS-extracting DES. And the compatibility of loaded DES with various PFAS destruction technologies (electrochemical, plasma, and SCWO) has been explored only in preliminary studies with limited analytical characterization of degradation by-products.

**Legal factors.** The regulatory treatment of DES components in drinking water falls into an uncharted category. Any detectable solvent residue in treated water will face stringent scrutiny from drinking-water regulators accustomed to evaluating inorganic and organic contaminants with established toxicological databases. For DES components without existing regulatory limits (which is the case for most terpene–fatty acid combinations), regulators may apply conservative default thresholds—potentially as low as the analytical LOQ—until substance-specific assessments are completed. Under REACH (Registration, Evaluation, Authorisation and Restriction of Chemicals), manufacturing or importing DES at quantities exceeding 1 tonne per year triggers registration obligations, including submission of physicochemical, toxicological, and ecotoxicological data packages. Proactive engagement with regulatory agencies, ideally through pre-submission consultations, and development of migration-testing protocols explicitly modelled on the food-contact materials framework (EU Regulation 10/2011) will be critical for establishing a viable regulatory pathway.

**Environmental factors.** From an environmental perspective, DES extraction must demonstrate life-cycle impacts that are at minimum equivalent to—and ideally better than—those of GAC and IX across multiple impact categories, as documented through the LCA framework proposed in Life-Cycle Assessment: Requirements, Hotspots, and Reporting Standards Section. A particular vulnerability is the sourcing of bio-based components: large-scale demand for menthol, thymol, or specific fatty acids could, if met through dedicated agricultural cultivation rather than waste-stream valorization, create land-use, water-use, and biodiversity trade-offs that undermine the ‘green’ narrative. Sourcing DES components from existing industrial waste streams (e.g., terpenes from citrus peel processing and fatty acids from vegetable oil refining residues) provides a far more environmentally defensible supply chain and should be prioritized wherever component purity specifications can be met.

### 4.2. SWOT Analysis

**Figure 5:**
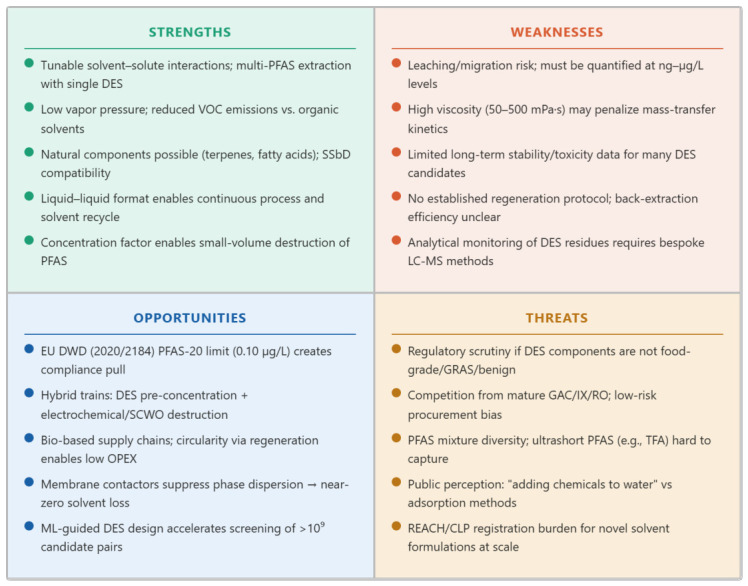
SWOT analysis for DES-based PFAS extraction technology, with five items per quadrant reflecting the current state of development (TRL 2–3) and the requirements for advancement toward pilot-scale demonstration (TRL 5–6).

Figure 5 presents a structured SWOT analysis that synthesizes the internal strengths and weaknesses of DES-based PFAS extraction technology alongside the external opportunities and threats identified through the PESTLE assessment. We elaborate on the most strategically significant elements of each quadrant below.

**Strengths.** The foremost strength of DES-based extraction lies in the extraordinary molecular tunability of the solvent system: by varying the identity, molar ratio, and combination of HBA and HBD components, the researcher can continuously adjust polarity, viscosity, interfacial tension, and specific interaction capacity to match the diverse physicochemical profiles of target PFAS. This tunability is unmatched by any single-material adsorbent and is the fundamental basis for the broad-spectrum extraction demonstrated in recent studies [22,23,24]. A second critical strength is the liquid–liquid extraction format itself, which enables continuous, steady-state operation with solvent recycle—a process paradigm that avoids the inherent batch characteristic of adsorption–desorption cycles and the associated capacity utilization losses. The pre-concentration function of LLE—collecting PFAS from large volumes of dilute water into a small volume of loaded DES—directly enables economically viable downstream destruction by operating on waste volumes that are orders of magnitude smaller than the original feed.

**Weaknesses.** The most consequential weakness is the inadequately characterized leaching behaviour of DES components at environmentally relevant concentrations. The literature to date reports extraction efficiencies and phase-separation characteristics but almost universally fails to report component-resolved aqueous-phase residuals at the ng/L–μg/L level using appropriately sensitive analytical methods. This information gap is not merely an academic lacuna—it represents the single largest technical barrier to regulatory acceptance. The characteristically high viscosities of many DES formulations (often 50–500 mPa·s at ambient temperature) impose mass-transfer limitations that may negate the thermodynamic advantages of high K_D_ values. Additionally, the absence of validated, multi-cycle regeneration data means that the practical cost of solvent replacement—and therefore the economic viability of the entire approach—remains essentially unknown beyond approximately 5–10 cycles.

**Opportunities.** The regulatory environment presents the most powerful external opportunity. With the EU PFAS-20 limit of 0.10 μg/L now legally binding and the US EPA MCLs entering their monitoring-and-compliance phase, the demand for effective, affordable PFAS treatment is not speculative but immediate and growing. DES extraction is particularly well-positioned to serve the emerging market for hybrid treatment trains—systems that combine pre-concentration (by DES-LLE) with downstream electrochemical or SCWO destruction—addressing the fundamental criticism that adsorption-based methods merely transfer PFAS from water to a solid waste stream. The rapid maturation of machine-learning approaches for molecular property prediction, combined with the vast combinatorial space of potential DES formulations (>10^5^ feasible binary pairs from bio-based component libraries), creates an unprecedented opportunity for accelerated computational screening that was not available even five years ago.

**Threats.** The most significant competitive threat is the continued incremental improvement of established technologies—particularly next-generation PFAS-selective ion-exchange resins that combine long operational lifetimes (>300,000 bed volumes) with single-use disposal to high-temperature incineration, eliminating the regeneration challenge entirely. If selective IX costs continue to decline through manufacturing scale-up, the economic window for DES-based alternatives may narrow. A second serious threat is the potential for negative public perception: the concept of adding a liquid chemical to drinking water, even one composed of food-grade ingredients, may encounter resistance in communities already sensitized to chemical contamination. Finally, the diversity of the PFAS contamination challenge itself—spanning long-chain legacy compounds, short-chain substitutes, ultrashort species like TFA, and emerging precursors—means that no single DES formulation may achieve universal applicability, potentially limiting market penetration to specific contamination profiles.

## 5. Actionable Recommendations and Screening Workflow

**Figure 6:**
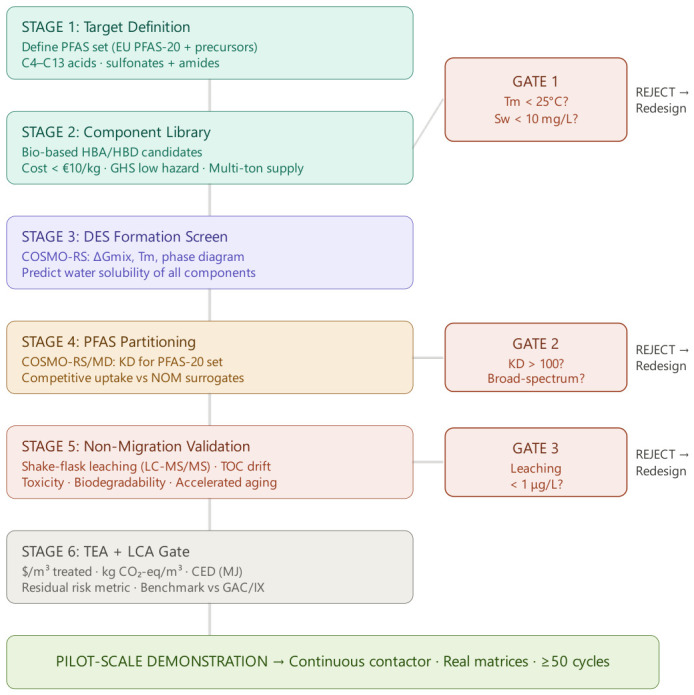
Proposed multi-stage screening workflow for PFAS-extracting DES development, linking molecular design to process demonstration through explicit go/no-go gates. Stage 1 defines the target PFAS set; Stage 2 generates the bio-based component library filtered by cost and hazard; Stage 3 screens DES formation and water solubility (Gate 1: Tm < 25 °C, aqueous solubility < 10mg/L); Stage 4 predicts and validates PFAS partitioning (Gate 2: KD > 100 for broad-spectrum coverage); Stage 5 validates non-migration at environmentally relevant concentrations (Gate 3: effluent leaching <1 μg/L per component); Stage 6 conducts TEA and LCA benchmarking against incumbent technologies. Candidates passing all gates advance to pilot-scale demonstration with continuous contactors and real water matrices over ≥50 cycles.

Drawing on the integrated analysis presented in Section 2, Section 3 and Section 4, we distil seven actionable recommendations that, if adopted collectively by the DES-for-PFAS research community, would substantially accelerate the transition from laboratory curiosities to deployable, commercially viable technology as well as to carry out suitable DES-screening processes, Figure 6.

(1) Treat non-migration as a quantitative performance metric from day one: Every publication reporting DES-based PFAS extraction should include component-resolved leaching data obtained by LC-MS/MS with LOQ ≤ 50 ng/L, TOC drift (ΔTOC after extraction), and pH drift (ΔpH) measured after extraction and after a minimum of 10 reuse cycles. Studies that report only percent mass recovery of the DES phase or visual confirmation of phase separation are insufficient for technology translation and should be clearly identified as preliminary.

(2) Screen against PFAS mixtures, not single compounds: The EU PFAS-20 list (C4–C13 perfluoroalkyl carboxylates and sulfonates) should serve as the minimum target compound set, supplemented by at least one neutral precursor or amide and, where analytically feasible, one ultrashort PFAS (TFA or PFPrA). All screening experiments should include realistic ionic strength (5–10 mM NaCl) and NOM (≥5 mg/L TOC as Suwannee River humic/fulvic acid or equivalent standardized reference material).

(3) Adopt multi-objective optimization as the default design paradigm: Simultaneous optimization can be achieved across ≥5 objectives (KD, viscosity, aqueous solubility, toxicity, component cost) using Pareto-front analysis. Publish the complete Pareto set, not only the single best-performing formulation, to enable community-wide learning and meta-analysis. Machine-learning models trained on curated DES physicochemical property databases can accelerate this screening by orders of magnitude beyond what is achievable by sequential experimental evaluation.

(4) Prefer process architectures that physically retain the DES phase@ For potable-water applications, membrane contactors or supported liquid phases should be the default contacting mode, given their inherent advantage in suppressing solvent carryover to diffusion-limited levels. Dispersive contactors (mixer–settlers and centrifugal extractors) may be acceptable for industrial side-stream or wastewater applications where residual DES concentration tolerances are orders of magnitude higher than for drinking water.

(5) Couple extraction with an explicit end-of-life strategy from the outset: Regeneration of the DES followed by destruction of a small, highly concentrated PFAS stream should be designed as an integral part of the process from the earliest stages, not deferred to ‘future work’. Candidate destruction methods (electrochemical oxidation, SCWO, and non-thermal plasma) must be evaluated for chemical compatibility with the DES matrix components and for the verified absence of toxic or persistent by-products.

(6) Establish and enforce minimum reporting standards All experimental studies should report complete DES composition (including residual water content, identity and concentration of impurities), density and dynamic viscosity at a minimum of two temperatures, phase behaviour characterization (visual + DSC), thermal and oxidative stability data, and reuse performance over ≥10 extraction–regeneration cycles with component-resolved analytical quality control at each cycle.

Tiered reporting set. To make the recommendations actionable at TRL 2–3, we now separate minimum Tier 1 metrics from more demanding Tier 2 indicators. Tier 1 should be reported by every early-stage study: PFAS removal/KD for a defined mixture, component-resolved DES leaching or a justified LOQ-limited surrogate, viscosity/water content after contact, and reuse over at least ten extraction–regeneration cycles. Tier 2 indicators, required before pilot advancement, include full PFAS-20 mixture testing, NOM/salinity stress tests, ≥50-cycle solvent-quality control, preliminary TEA/LCA inventories, by-product analysis after destruction, and membrane-contactor or centrifugal-contactor operation with real matrices.

(7) Benchmark cost and environmental impacts early using transparent, simplified TEA/LCA. Even at TRL 2–3, researchers can and should compile mass and energy balances and estimate unit treatment costs ($/m^3^) and key environmental impacts (kg CO_2_-eq/m^3^, CED in MJ/m^3^) using the framework presented in Table 3 and Section Life-Cycle Assessment: Requirements, Hotspots, and Reporting Standards. Solvent make-up cost, analytical monitoring cost, and waste-handling cost must all be included. Studies that claim ‘green’ or ‘sustainable’ credentials without any quantitative LCA or TEA data should be viewed with appropriate scientific scepticism.

## 6. Conclusions and Outlook

Hydrophobic deep eutectic solvents and NADESs have crossed the threshold [18,19,20,21,22,23,24] from conceptual ‘green solvents’ to credible PFAS extractants, with recent demonstrations achieving > 99% removal efficiencies for multiple PFAS classes [22,24] in real-water matrices, mechanistic understanding at the atomistic level through advanced computational methods (COSMO-RS, AIMD) [19,20,23,30], and initial—though still preliminary—explorations of integrated capture-and-destroy platforms. Yet the pathway from these encouraging laboratory results to real-world deployment remains constrained by three critical and interconnected knowledge gaps: (i) the absence of systematic structure–leaching relationships for DES components at the environmentally relevant ng/L–μg/L concentration range; (ii) the lack of validated regeneration and solvent-quality-control protocols demonstrated over industrially relevant cycle numbers (≥50, ideally >200); and (iii) insufficient techno-economic and life-cycle benchmarking against the mature, extensively cost-optimized incumbent technologies that any new entrant must outperform.

We argue that the field should now pivot decisively toward three strategic research directions. First, the bifunctional DES design concept should be systematically adopted—decoupling fluorophilic tail solvation from polar headgroup capture using complementary bio-based components—as the unifying molecular framework for both experimental and computational screening campaigns. Second, non-migration should be elevated from an afterthought to a first-class, quantitative design objective with explicit numerical specifications analogous to those governing food-contact polymer migration, enforced through rigorous component-resolved analytical protocols. Third, DES molecular design should be coupled early and mandatorily to scalable process architecture selection (with membrane contactors as the default for potable-water applications) and validated destruction pathways for the concentrated PFAS waste stream.

The highest-impact near-term research priorities are: (a) systematic mapping of molecular structure–aqueous solubility–leaching relationships across terpene, fatty acid, and natural alkaloid component families, incorporating the effects of water content, pH, ionic strength, and the presence of NOM and common co-contaminants; (b) development and validation of mixture-aware PFAS extraction models that account for competitive uptake by NOM and the dependence of distribution coefficients on ionic strength and temperature; (c) pilot-scale demonstrations at the 100–1000 L/h scale using membrane contactors or centrifugal extractors coupled to on-site electrochemical or SCWO destruction of concentrated PFAS eluates; and (d) machine-learning-accelerated screening [38,39] of the vast DES combinatorial design space (>105 feasible binary pairs from bio-based component libraries) using Gaussian process regression or graph neural network models [38,39] trained on systematically curated experimental databases.

If these research priorities are pursued with the transparency, rigour, and quantitative benchmarking advocated throughout this perspective—and if accompanied by proactive engagement with drinking-water regulators on novel solvent approval pathways—DES-based liquid–liquid extraction has a credible pathway to becoming a modular ‘PFAS concentrator’ technology. Such a technology would be particularly valuable for three target applications: small- and medium-sized water utilities facing disproportionate compliance costs under new regulations, industrial side-streams and landfill leachates with elevated PFAS concentrations (ng/L to μg/L range) and managed waste-discharge requirements, and as a pre-concentration step enabling energy-efficient thermal or electrochemical destruction of PFAS in minimal volumes. Ultimately, PFAS-extracting DES should not be evaluated as standalone solvents but as integrated, closed-loop treatment materials: a candidate merits pilot study advancement only when it simultaneously passes four coupled tests—broad mixture-level PFAS affinity across the target compound set, verified non-migration at ng/L–µg/L resolution, stable regeneration coupled to effective PFAS destruction, and quantified cost and environmental competitiveness relative to incumbent processes—under the intended water matrix.

The EU 2026 compliance deadline and the escalating global regulatory momentum define the window of opportunity. This 3–5 year horizon is not arbitrary: it reflects the convergence of the first major utility procurement cycles triggered by the new PFAS limits, the time typically required to progress from TRL 2–3 formulation studies through validated pilot demonstration at TRL 5–6, and the pace at which next-generation selective ion-exchange resins, advanced adsorbents, and membrane processes continue to improve. DES-based PFAS extraction must demonstrate stable solvent retention, closed-loop regeneration, and concentrate destruction at pilot scale within this window, or risk being introduced too late to compete with technologies that, although imperfect, are already being installed.

## Figures and Tables

**Table 1:** Minimum design criteria for PFAS-extracting DES: quantitative targets and validation methods.

Criterion	Rationale	Target	Validation	Evidence Status/Threshold Logic
Broad PFAS affinity	Must remove PFCAs, PFSAs, and amides across C4–C13	KD > 100 for ≥15 of EU PFAS-20; enrichment factor > 50	COSMO-RS/MD screening; batch LLE with PFAS-20 at 1 μg/L; isotherms	Partly demonstrated for selected long-chain targets; KD > 100 is a prospective broad-mixture gate enabling compact enrichment, not a universal compliance guarantee.
Non-migration	Potable standards require ng–μg/L residuals; solvent loss drives OPEX	Aq. solubility < 10 mg/L; effluent < 1 μg/L per component; ΔpH < ±0.3	Shake-flask; TOC; component-resolved LC-MS/MS; 50-cycle ageing	Engineering target. The <1 µg/L effluent value is a conservative non-migration specification, not a current DES-specific regulatory limit.
Low viscosity	Controls contactor size/energy; viscous DES kinetically limited	η < 50 mPa·s at 25–40 °C; kLa > 0.01 s^−1^	Rheometry; pendant drop; mass-transfer coefficient in bench contactor	Achieved by some low-viscosity hydrophobic DES families; threshold reflects mass-transfer and contactor-energy constraints.
Benign and scalable	Regulatory acceptance; cost depends on hazard and supply	Food-grade/GRAS; OECD 301 biodegradable; <€10/kg	GHS classification; biodegradability tests; supplier surveys; LCA	Prospective SSbD and supply-chain target; must be confirmed by GHS/OECD/REACH-type evidence.
Thermal stability	Long-term cycling; avoid degradation products	No decomposition after 30 d at 40 °C; TGA onset > 150 °C	Accelerated stability; TGA/DSC; GC-MS headspace	Usually achievable for terpene/fatty-acid systems; must be verified after hydration and repeated regeneration.
Regenerability	Closed-loop essential for cost and sustainability	PFAS back-extraction > 90%/cycle; DES recovery > 98%; stable KD over ≥50 cycles	Back-extraction with alkaline brine; NMR/FT-IR quality control	Partly demonstrated over limited cycles; ≥50 cycles is the minimum translational target for pilot relevance.

**Table 2:** Illustrative application of the multi-criteria screening checklist to representative DES-PFAS extraction concepts.

Candidate/Evidence Basis	Bifunctional Architecture	Extraction Evidence	Checklist Outcome	Principal Gap Before Advancement
Menthol:acetic acid NADES [18]	Hydrophobic menthol domain; weak/small acidic HBD	Proof-of-concept PFHpA extraction	Fails non-migration risk because acetic acid is water-miscible	Replace short acid with hydrophobic HBD/HBA and quantify component leaching
TOPO:lauric acid DES [21]	Strong P=O HBA plus C12 hydrophobic acid domain	Rapid, high PFOA removal and reuse reported	Passes affinity gate for model long-chain PFAS; not natural/SSbD-complete	Demonstrate PFAS-20 mixtures, short-chain uptake, and ng/L component residuals
Ionizable DES for neutral/ionizable PFAS [22,23]	Hydrophobic domain plus ionizable/basic binding sites	Multi-class PFAS removal and AIMD-supported motifs	Promising broad-spectrum concept	Quantify leaching, NOM competition, regeneration, and by-products
Matrine-based low-melting mixture [28]	Bio-based alkaloid HBA/basic site embedded in bulky hydrophobic scaffold	Computationally promising for PFOA extraction	Potential SSbD lead; experimental status incomplete	Validate DES formation, viscosity, KD, and effluent migration experimentally

**Table 3:** Techno-economic framework for DES-based PFAS extraction: key cost levers, target ranges, and benchmark comparison.

Cost Lever	DES Target	GAC Benchmark	IX Benchmark	Sensitivity
Solvent cost (€/kg)	5–15	1.5–3.0	50–200	Bio-sourcing varies ±100%
S:W ratio (*v*/*v*)	1:10–1:50	N/A	N/A	Drives equipment size
Make-up (%/cycle)	<2%	100%	100%	Dominant OPEX driver
Total OPEX ($/m^3^)	0.10–0.40	0.15–0.80	0.10–0.60	Regeneration energy dominates
CAPEX ($/m^3^ ann.)	0.05–0.15	0.10–0.25	0.05–0.15	Membrane contactors add cost
Monitoring ($/m^3^)	0.02–0.05	0.01–0.03	0.01–0.03	Bespoke LC-MS adds cost

**Table 4:** PESTLE analysis for DES-based PFAS extraction: external factors, implications, and recommended strategic responses.

Factor	Implications	Recommended Response
Political	EU DWD PFAS limits (2026) and US EPA MCLs (2024) create strong compliance pull; EU PFAS restriction proposal (~10,000 substances) may further expand demand; funding programmes prioritize deployable, low-risk solutions	Align DES development with regulatory timelines; target EU-funded demonstration projects (Horizon Europe, LIFE); engage with regulatory agencies on novel solvent approval pathways through pre-submission consultations
Economic	Small/medium utilities face disproportionate per capita costs due to economies of scale in GAC/IX; solvent make-up and bespoke analytical monitoring are critical OPEX sensitivities; modular units preferred for distributed treatment	Develop low-cost, containerized DES extraction units for <10,000 PE systems; minimize solvent losses to <0.5%/cycle through membrane contactors; establish cost-sharing models for regional analytical monitoring
Social	Public acceptance critically depends on ‘no new chemicals in my water’ perception; community engagement and transparent third-party toxicity data are prerequisites for social licence to operate	Use exclusively food-grade/GRAS components; publish toxicity and biodegradability data in open access; develop plain-language communication materials explaining the extraction concept
Technological	Mass-transfer limitations from high viscosity; NOM fouling in real matrices uncharacterized; regeneration/destruction validation lacking; by-product formation unknown for most DES–destruction combinations	Prioritize low-viscosity formulations (η < 50 mPa·s); develop anti-fouling strategies; validate over ≥50 cycles; characterize destruction by-products by LC-HRMS and ion chromatography (F^−^)
Legal	Any solvent residue in treated water faces stringent scrutiny; REACH registration required at >1 ton/year; drinking-water regulators lack established assessment frameworks for DES components	Proactively compile REACH-compliant dossiers for lead DES; establish migration testing analogous to EU Reg 10/2011; pursue food-grade certification; engage regulators through pre-submission consultations
Environmental	Life-cycle impacts must match or exceed GAC/IX across multiple categories; bio-based sourcing may create land-use and biodiversity trade-offs; aquatic ecotoxicity of leaked DES components remains uncharacterized	Conduct comparative LCA from TRL 3 onwards; source components from industrial waste streams where possible; include aquatic ecotoxicity and marine sediment toxicity in screening criteria

## Abbreviations

PFASs	Per- and polyfluoroalkyl substances
DESs	Deep eutectic solvents
NADESs	Natural deep eutectic solvents
HBA	Hydrogen bond acceptor
HBD	Hydrogen bond donor
GAC	Granular activated carbon
IX	Ion exchange
NF/RO	Nanofiltration/reverse osmosis
LLE	Liquid–liquid extraction
NOM	Natural organic matter
PFCAs	Perfluoroalkyl carboxylic acids
PFSAs	Perfluoroalkyl sulfonic acids
TFA	Trifluoroacetic acid
PFOA	Perfluorooctanoic acid
PFOS	Perfluorooctanesulfonic acid
PFHxS	Perfluorohexanesulfonic acid
PFNA	Perfluorononanoic acid
HFPO-DA	Hexafluoropropylene oxide dimer acid
MCL	Maximum contaminant level
NPDWR	National Primary Drinking Water Regulation
DWD	Drinking Water Directive
SSbD	Safe-and-sustainable-by-design
COSMO-RSs	Conductor-like screening model for real solvents
MDs	Molecular dynamics
AIMDs	Ab initio molecular dynamics
ML	Machine learning
TEA	Techno-economic analysis
LCA	Life-cycle assessment
CAPEX	Capital expenditure
OPEX	Operational expenditure
TRL	Technology readiness level
GRAS	Generally recognized as safe
REACH	Registration, evaluation, authorisation and restriction of chemicals
GHS	Globally harmonized system
LC-MS/MS	Liquid chromatography–tandem mass spectrometry
LC-HRMS	Liquid chromatography–high-resolution mass spectrometry
LOQ	Limit of quantification
TOC	Total organic carbon
SCWO	Supercritical water oxidation
CED	Cumulative energy demand
TGA	Thermogravimetric analysis
DSC	Differential scanning calorimetry
NMR	Nuclear magnetic resonance
FT-IR	Fourier-transform infrared spectroscopy

## References

[1] OECD (2021). Reconciling Terminology of the Universe of Per- and Polyfluoroalkyl Substances: Recommendations and Practical Guidance.

[2] Wang Z., DeWitt J.C., Higgins C.P., Cousins I.T. (2017). A never-ending story of per- and polyfluoroalkyl substances (PFASs)?. Environ. Sci. Technol..

[3] O’Hagan D. (2008). Understanding organofluorine chemistry. An introduction to the C–F bond. Chem. Soc. Rev..

[4] European Union (2020). Directive (EU) 2020/2184 of the European Parliament and of the Council of 16 December 2020 on the quality of water intended for human consumption (recast). Off. J. Eur. Union.

[5] European Commission (2026). New EU-Wide Protections Against PFAS in Drinking Water Come into Effect. https://environment.ec.europa.eu/news/new-eu-rules-limit-pfas-drinking-water-2026-01-12_en.

[6] U.S. EPA (2024). Final PFAS National Primary Drinking Water Regulation.

[7] Arp H.P.H., Gredelj A., Glüge J., Scheringer M., Cousins I.T. (2024). The global threat from the irreversible accumulation of trifluoroacetic acid (TFA). Environ. Sci. Technol..

[8] Neuwald I.J., Hübner D., Wiegand H.L., Valkov V., Borchers U., Nödler K., Scheurer K., Hale S.E., Arp H.P.H., Zahn D. (2022). Ultra-short-chain PFASs in the sources of German drinking water: Prevalent, overlooked, difficult to remove, and unregulated. Environ. Sci. Technol..

[9] ECHA (2023). Annex XV Restriction Report: Proposal for a Restriction of Per- and Polyfluoroalkyl Substances (PFASs).

[10] (2024). Technologies and Cost for Removing Per- and Polyfluoroalkyl Substances (PFAS) from Drinking Water.

[11] Gagliano E., Sgroi M., Falciglia P.P., Vagliasindi F.G.A., Roccaro P. (2020). Removal of poly- and perfluoroalkyl substances (PFAS) from water by adsorption: Role of PFAS chain length, effect of organic matter, and challenges in adsorbent regeneration. Water Res..

[12] Appleman T.D., Higgins C.P., Quiñones O., Vanderford B.J., Kolstad C., Zeigler-Holady J.C., Dickenson E.R.V. (2014). Treatment of poly- and perfluoroalkyl substances in U.S. full-scale water treatment systems. Water Res..

[13] Boyer T.H., Fang Y., Ellis A., Dietz R., Choi Y.J., Schaefer C.E., Higgins C.P., Strathmann T.J. (2021). Anion exchange resin removal of per- and polyfluoroalkyl substances (PFAS) from impacted water: A critical review. Water Res..

[14] Stoiber T., Evans S., Naidenko O.V. (2020). Disposal of products and materials containing per- and polyfluoroalkyl substances (PFAS): A cyclical problem. Chemosphere.

[15] Winchell L.J., Ross J.J., Wells J.M., Fonoll X., Norton J.W., Bell K.Y. (2021). Per- and polyfluoroalkyl substances thermal destruction at water resource recovery facilities: A state-of-the-science review. Water Environ. Res..

[16] Abbott A.P., Capper G., Davies D.L., Rasheed R.K., Tambyrajah V. (2003). Novel solvent properties of choline chloride/urea mixtures. Chem. Commun..

[17] van Osch D.J.G.P., Zubeir L.F., van den Bruinhorst A., Rocha M.A.A., Kroon M.C. (2015). Hydrophobic deep eutectic solvents as water-immiscible extractants. Green Chem..

[18] Fortunato L., Al Fuhaid L., Murgolo S., De Ceglie C., Mascolo G., Falivene L., Vrouwenvelder J.S., Witkamp G.J., Farinha A. (2023). Removal of PFAS from water using hydrophobic NADES: A proof-of-concept study. J. Water Process Eng..

[19] Eid S., Darwish A.S., Lemaoui T., Banat F., Hasan S.W., AlNashef I.M. (2023). Multicriteria design of novel natural hydrophobic deep eutectic solvents for PFAS extraction using COSMO-RS. J. Mol. Liq..

[20] Gutiérrez A., Maletta A., Aparicio S., Atilhan M. (2023). A theoretical study of low-concentration PFAS remediation by novel hydrophobic deep eutectic solvents. J. Mol. Liq..

[21] Eid S.M., Lemaoui T., Darwish A.S., Jaoude M.A.A., Banat F., Hasan S.W., AlNashef I.M. (2024). Rapid and highly efficient removal of aqueous PFOA using deep eutectic solvents. Chem. Eng. J..

[22] Fan C., Cheng L., Deng W. (2024). Design of deep eutectic solvents for multiple PFAS removal: Energy-based screening and mechanism elucidation. Sci. Total Environ..

[23] Fan C., Shan Y., Cheng L., Yin Y. (2025). Endowing deep eutectic solvents with neutral and ionizable PFAS extracting capability: Experimental study and AIMD simulation. Green Chem. Lett. Rev..

[24] Chen J., Bentel M.J., Niu X.Z. (2025). Hydrophobic deep eutectic solvent platform for integrated PFAS capture and in situ mild-condition mineralization. ACS Sustain. Chem. Eng..

[25] Devi M., Moral R., Thakuria S., Mitra A., Paul S. (2023). Hydrophobic deep eutectic solvents as greener substitutes for conventional organic solvents in extraction. ACS Omega.

[26] Ishtaweera P., Baker G.A. (2024). Progress in the application of ionic liquids and deep eutectic solvents for the separation and quantification of PFAS. J. Hazard. Mater..

[27] Klamt A., Eckert F. (2001). COSMO-RS: A novel and efficient method for the a priori prediction of thermophysical data of liquids. Fluid Phase Equilib..

[28] Martel S., Aguilar N., Gutiérrez A., Rozas S., Marcos P.A., Bol A., Atilhan M., Trenzado J.L., Aparicio S. (2025). Computational study of eco-friendly PFOA extraction via matrine-based low melting mixture. J. Mol. Liq..

[29] European Commission (2011). Commission Regulation (EU) No 10/2011 on plastic materials and articles intended to come into contact with food. Off. J. Eur. Union.

[30] Klamt A. (1995). Conductor-like screening model for real solvents: A new approach to the quantitative calculation of solvation phenomena. J. Phys. Chem..

[31] Alonso D.A., Baeza A., Chinchilla R., Guillena G., Pastor I.M. (2016). Deep eutectic solvents: The organic reaction medium of the century. Eur. J. Org. Chem..

[32] Awaja N.E., Almuistafa G., Darwish A.S., Lemaoui A.S., Benguerba Y., Banat F., Arafat H.A., AlNashef I. (2023). Molecular-based artificial neural networks for selecting deep eutectic solvents for the removal of contaminants from aqueous media. Chem. Eng. J..

[33] Abbas U.L., Zhang Y., Tapia J., Md S., Chen J., Shi J., Shao Q. (2024). Machine-Learning-Assisted Design of Deep Eutectic Solvents Based on Uncovered Hydrogen Bond Patterns. Engineering.

[34] Ellis A.C., Boyer T.H., Fang Y., Liu C.J., Strathmann T.J. (2023). Life cycle assessment and life cycle cost analysis of anion exchange and granular activated carbon for PFAS remediation. Water Res..

[35] (2006). Environmental Management—Life Cycle Assessment—Requirements and Guidelines.

[36] Gabelman A., Hwang S.-T. (1999). Hollow fiber membrane contactors. J. Membr. Sci..

[37] Song X., Montelius M., Carlsson C. (2024). Life Cycle Assessment of Per- and Polyfluoroalkyl Substances (PFAS) Remediation Technologies: A Literature Review. Environments.

[38] Abbott A.P., Boothby D., Capper G., Davies D.L., Rasheed R.K. (2004). Deep eutectic solvents formed between choline chloride and carboxylic acids. J. Am. Chem. Soc..

[39] Glüge J., Scheringer M., Cousins I.T., DeWitt J.C., Goldenman G., Herzke D., Lohmann R., Ng C.A., Trier X., Wang Z. (2020). An overview of the uses of per- and polyfluoroalkyl substances (PFAS). Environ. Sci. Process. Impacts.

